# Postpartum diabetes screening among low income women with gestational diabetes in Missouri 2010–2015

**DOI:** 10.1186/s12889-019-6475-0

**Published:** 2019-02-04

**Authors:** Cynthia J. Herrick, Matthew R. Keller, Anne M. Trolard, Ben P. Cooper, Margaret A. Olsen, Graham A. Colditz

**Affiliations:** 1 Division of Endocrinology, Metabolism and Lipid Research, Department of Medicine, Washington University School of Medicine, 660 S. Euclid Ave, Campus Box 8127, St. Louis, MO 63110 USA; 2 Division of Public Health Sciences, Department of Surgery, Washington University School of Medicine, 660 S. Euclid Ave, Campus Box 8100, St. Louis, MO 63110 USA; 3 Center for Administrative Data Research, Washington University School of Medicine, 4523 Clayton Ave, CB 8051, St. Louis, MO 63110 USA; 4 Public Health Data and Training Center, Institute for Public Health, Washington University School of Medicine, 660 S. Euclid Ave, Campus Box 8217, St. Louis, MO 63110 USA; 5 Centene Corporation, 7700 Forsyth Blvd, St. Louis, MO 63105 USA

**Keywords:** Gestational diabetes, Postpartum screening, Care transition, Healthcare access, Medicaid

## Abstract

**Background:**

Gestational diabetes increases risk for type 2 diabetes seven-fold, creating a large public health burden in a young population. In the US, there are no large registries for tracking postpartum diabetes screening among women in under-resourced communities who face challenges with access to care after pregnancy. Existing data from Medicaid claims is limited as women often lose this coverage within months of delivery. In this study, we aim to leverage data from electronic health records and administrative claims to better assess postpartum diabetes screening rates among low income women.

**Methods:**

A retrospective population of 1078 women with gestational diabetes who delivered between 1/1/2010 and 10/8/2015 was generated by linking electronic health record data from 21 Missouri Federally Qualified Health Centers (FQHCs) with Medicaid administrative claims. Screening rates for diabetes were calculated within 12 weeks and 1 year of delivery. Initial screening after the first postpartum year was also documented.

**Results:**

Median age in the final population was 28 (IQR 24–33) years with over-representation of black non-Hispanic and urban women. In the final population, 9.7% of women had a recommended diabetes screening test within 12 weeks and 18.9% were screened within 1 year of delivery. An additional 125 women received recommended screening for the first time beyond 1 year postpartum. The percentage of women who had a postpartum visit (83.9%) and any glucose testing (40.6%) in the first year far exceeded the proportion of women with recommended screening tests.

**Conclusions:**

Linking electronic health record and administrative claims data provides a more complete picture of healthcare follow-up among low income women after gestational diabetes. While screening rates are higher than reported with claims data alone, there are opportunities to improve adherence to screening guidelines in this population.

**Electronic supplementary material:**

The online version of this article (10.1186/s12889-019-6475-0) contains supplementary material, which is available to authorized users.

## Introduction

More than half of women with gestational diabetes develop type 2 diabetes, with most cases occurring within 5–10 years of the index pregnancy [1, 2]. Additionally, gestational diabetes prevalence is highest among racial and ethnic minorities and women of lower socioeconomic status [3]. Furthermore, while Asian women are more likely to have gestational diabetes, black and Hispanic women are more likely to progress to type 2 diabetes [4]. Following gestational diabetes, both the American Diabetes Association (ADA) and the American Congress of Obstetricians and Gynecologists (ACOG) recommend screening for type 2 diabetes at 4–12 weeks postpartum with a fasting plasma glucose (FPG) or 2 h oral glucose tolerance test (oGTT) and screening with either of these tests or a hemoglobin A1C (HbA1C) every 1–3 years thereafter [5, 6].

Existing data on screening rates among low income women are limited. A study of 6239 women utilizing South Carolina Medicaid claims data suggested that screening with a recommended test (FPG or oGTT) occurred in only 3.2% of cases between 5 and 13 weeks postpartum [7]. Another claims data study including 2367 women on Medicaid in Maryland found screening with any glucose test occurred in 5.7% of women by 12 weeks and 15.2% by 1 year [8]. These studies likely underestimate screening in this population, given variable Medicaid income eligibility criteria before, during, and after pregnancy. In Missouri, many women become eligible for Medicaid during pregnancy but lose comprehensive coverage 60 days after delivery. In fact, most existing data in the US on screening for type 2 diabetes after a pregnancy with gestational diabetes come from large integrated health systems, single centers, and claims data [7–14]. Fragmentation of care between health systems prevents follow-up of screening over time. Additionally, women receive prenatal and postpartum care in clinics and often deliver in hospitals. Assessing screening time frames post-delivery is difficult if inpatient and outpatient systems are not integrated. Moreover, there are no published data on screening for type 2 diabetes that occurs beyond the first postpartum year.

To better understand healthcare utilization and screening among low income women, addressing a critical gap in the literature, we sought to leverage the strengths of electronic health record (EHR) data and Medicaid claims data to create a unique linked dataset with information on prenatal care through delivery into the postpartum period. We did not limit our data collection to only the first postpartum year, and we incorporated variables describing the neighborhood food and built environment that could have relevance to diabetes screening and prevention. In this manuscript, we describe our procedure for creating this novel data resource and report population estimates for postpartum screening according to guideline recommendations.

## Methods

Data from most Federally Qualified Health Centers (FQHC) in Missouri flow into a central data repository. Health centers that are members of the Missouri Primary Care Association (MPCA) use different EHR systems, thus all data enters a data warehouse, managed by Azara Healthcare, and is mapped to standardize reporting across fields. Our study was reviewed and approved by the Washington University Human Research Protection Office in October 2015. The Data Use Agreement with the MPCA was fully executed in April 2016, defining how the data would be used and specifying precautions for storing and transferring the data securely. Twenty six of 29 health center systems gave permission for their data to be utilized. Data in the final population of women with gestational diabetes came from 21 health center systems.

**Fig. 1:**
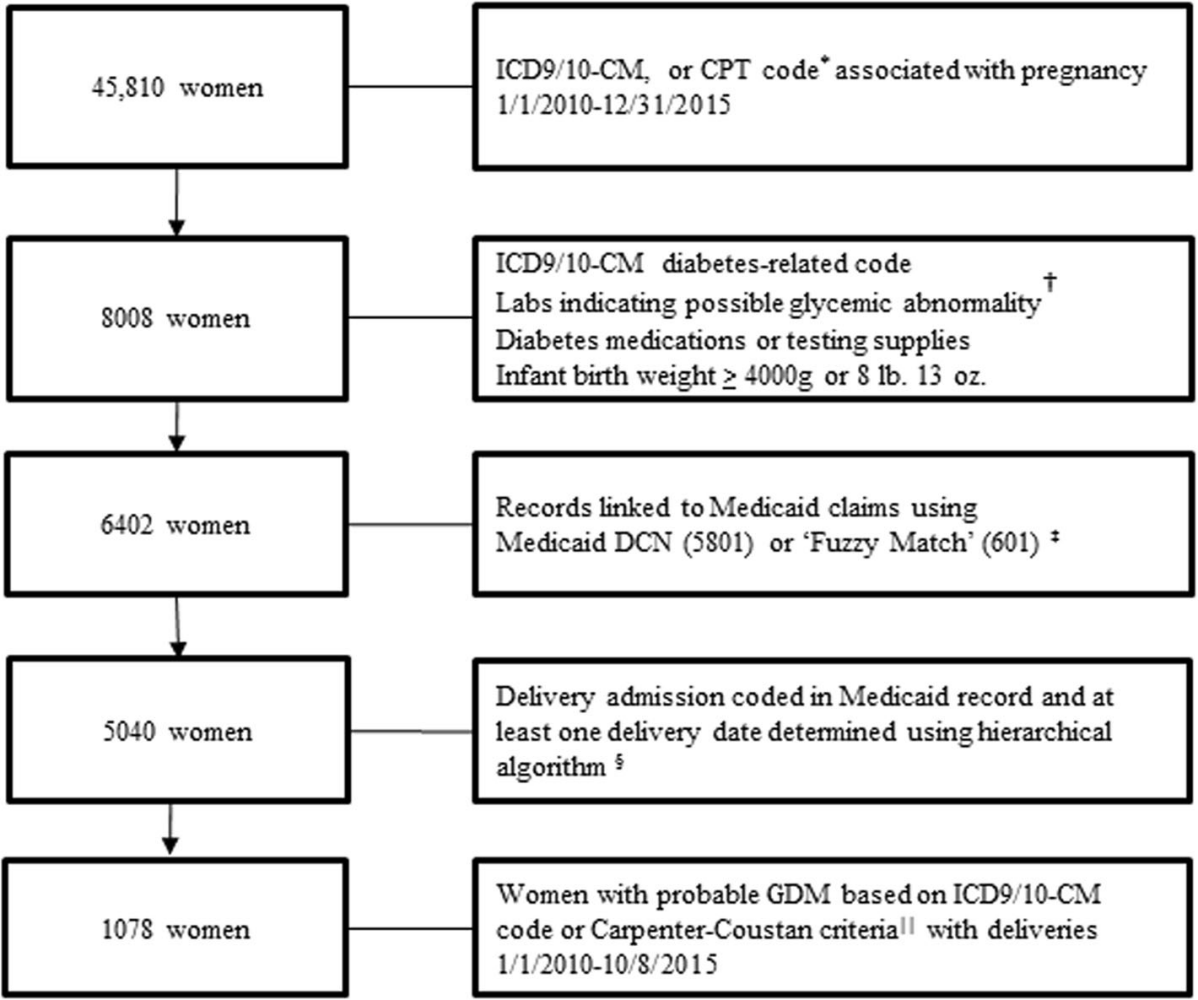
Creation of the linked Medicaid-EHR retrospective study population. ^*^ ICD9: 640–679, V22-V27, V91; ICD10: O09, Z34, Z37, Z39, O10-O16, O20-O26, O28-O36, O40-O48, O60-O77, O80, O82, O85, O86, O87-O92, O94, O98-O99, O9A, Z3A, A34; CPT: 59400, 59409, 59410, 59412, 59414, 59425, 59426, 59430, 59510, 59514, 59515, 59525, 59610, 59612, 59614, 59618, 59620, 59622, 76801, 76802, 76805, 76810–76821, 76825-76828, 59025. ^†^ One or more: fasting glucose > 5.27 mmol/L, Glucose Challenge Test or single 1 h glucose > 7.21 mmol/L, 1 h glucose > 9.99 mmol/L if a 2 and 3 h glucose were recorded on same day, 2 h glucose > 8.6 mmol/L, 3 h glucose > 7.77 mmol/L, A1C > 6.0% (42 mmol/mol), random glucose > 7.21 mmol/L, Any + urine glucose. ^‡^ Fuzzy match utilized first name, last name, date of birth, zip code and phone number to match Azara record with Medicaid record if no Medicaid identifier available. ^§^Algorithm to identify deliveries: 1) facility and provider claims with CPT-4 or ICD 9/10 code for delivery during an inpatient admission; 2) CPT-4 or ICD9/10 code for delivery and an additional code for delivery-related diagnosis or procedure; 3) CPT-4 or ICD 9/10 code for delivery and a provider claim for anesthesia/epidural; 4) CPT-4 or ICD9/10 code for delivery and a provider claim for postpartum care following birth; or 5) 3 or more delivery-specific codes (including pathologic examination of the placenta and revenue code for labor room (720–724, 729)). ^||^ This is the strictest definition for inclusion: ICD-9/10CM code for gestational diabetes in pregnancy (648.8x, O24.4x) or 2 or more abnormal values on 3 h oGTT by Carpenter and Coustan Criteria: Fasting glucose > 5.27 mmol/L, 1 h glucose > 9.99 mmol/L, 2 h glucose > 8.6 mmol/L, 3 h glucose > 7.77 mmol/L. Deliveries after 10/8/2015 were excluded to allow everyone the opportunity for 84 days (12 weeks) post-delivery records

Figure 1 provides an overview of the inclusion criteria for the population definition procedure. In July 2016, an initial de-identified dataset was transferred from Azara Healthcare to investigators at Washington University. Azara Healthcare generated random number IDs for each patient in this dataset and included any woman with an International Classification of Diseases, Clinical Modifications versions 9 or 10 (ICD-9/10-CM) diagnosis code or Current Procedural Terminology, 4th edition (CPT-4) code related to pregnancy care in her record between January 1, 2010 and December 31, 2015. Files contained age, race, ethnicity, primary language, patient encounters (date, time, provider name and type (if documented), health center, vital signs, height, weight), charges, payer information, medications and supplies, laboratory data, infant birthweight, and delivery date (if available). However, the clinic EHR data was incomplete in regard to delivery date as well as administrative codes for pregnancy comorbidities and delivery complications.

Therefore, we linked EHR data with Medicaid claims to obtain additional data on pregnancy comorbidities and delivery complications and to define an accurate delivery date for assessing postpartum screening time frames and marking the gestational period. First, we defined ‘potential gestational diabetes candidates’ utilizing criteria for abnormal glucose. This was intentionally broad to be most sensitive in identifying all potential individuals with glucose abnormalities who may have had gestational diabetes during pregnancy. Criteria for the gestational diabetes candidate subset (8008 women) are defined in Fig. 1.

**Fig. 2:**
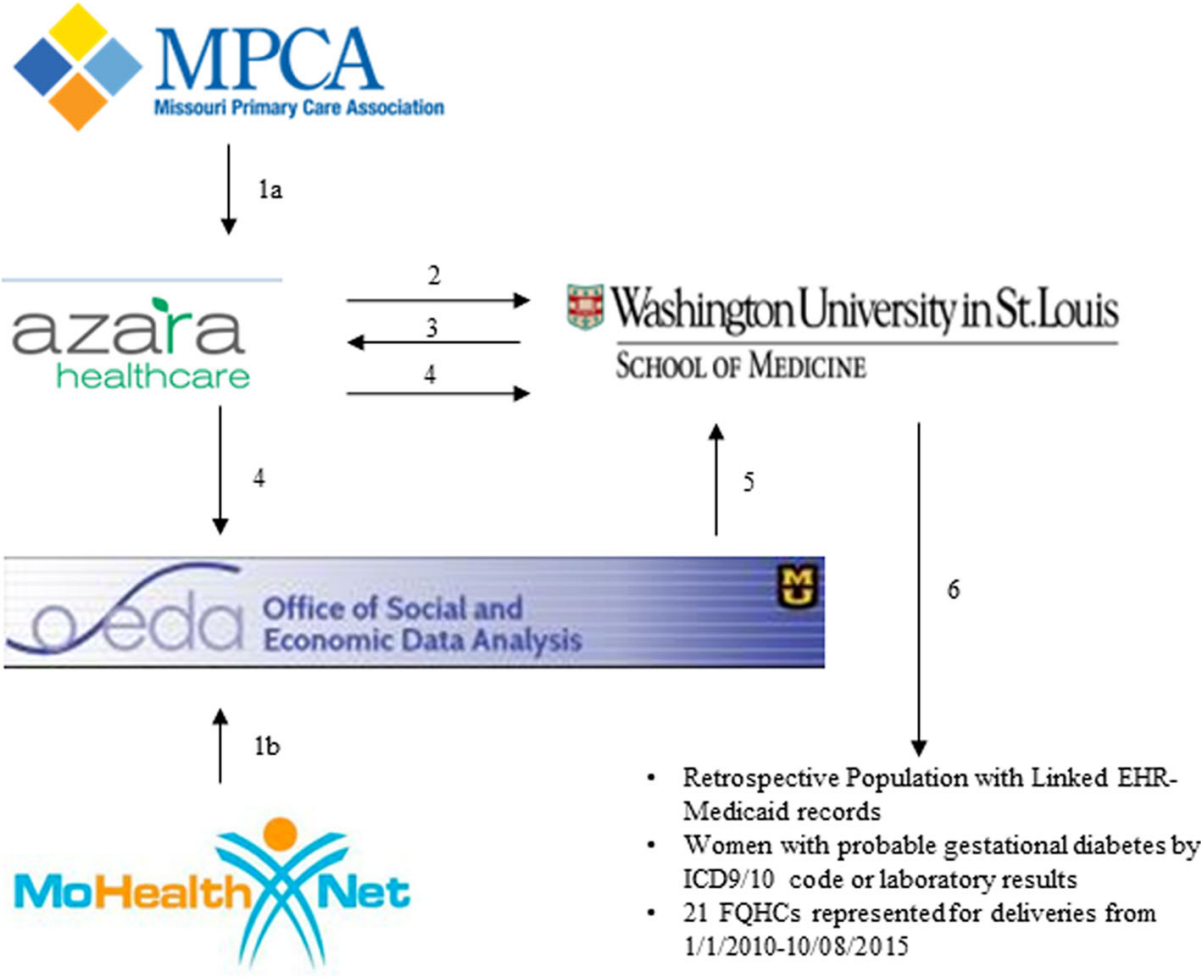
Diagram of flow and type of data from each source. 1a) EHR data flows from MPCA member health centers to central data repository with Azara Healthcare; 26 health centers approved use of data for this study. 1b) Claims data flows from Missouri Medicaid (fee for service and managed care plans) to the Office of Social and Economic Data Analysis (OSEDA). 2) De-identified EHR data from women with pregnancy related codes 1/1/2010–12/31/2015 sent to Washington University (demographics, encounter data, charges, payer information, medications/supplies ordered, laboratory data, infant birthweight (if available), and delivery date (if available) - delivery date and codes related to comorbidities and complications were limited. 3) Potential gestational diabetes candidates identified based on combination of ICD-9/10-CM codes, medication and supply data, laboratory data, and infant birthweight – randomly generated number ID for potential gestational diabetes candidates transferred back to Azara Healthcare. 4) Azara Healthcare transferred Medicaid identifier, first name, last name, date of birth, zip code and phone number with randomly generated number ID to OSEDA for linking with claims data from ‘potential gestational diabetes candidates’ with delivery date 1/1/2010–12/31/2015; Address on these individuals was transferred separately to Washington University for creation of geographic variables. 5) OSEDA transferred randomly generated number ID with linked inpatient and outpatient claims to Washington University (included medical eligibility code, place of service code, revenue codes, provider specialty code, National Provider Identifier, provider type code, and dates of service with ICD-9/10-CM diagnosis, ICD-9-CM/ICD-10-Procedure Coding System (PCS) procedure codes, and CPT-4 codes). 6) Washington University narrows the population to women with probable gestational diabetes defined by ICD-9/10-CM code or Carpenter and Coustan laboratory criteria based on defining delivery date, period of gestation, and postpartum period

We further narrowed the gestational diabetes candidate subset to those with probable gestational diabetes using claims data. The random number IDs for each patient in the gestational diabetes candidate subset were returned to Azara Healthcare. Identifiers on this subset were securely transferred from Azara Healthcare to the Office of Social and Economic Data Analysis (OSEDA) at the University of Missouri (the state contractor for Medicaid data) for linkage with Medicaid claims data for each individual. Claims data included inpatient and outpatient claims with medical eligibility code, place of service code, revenue codes, provider specialty code, National Provider Identifier, provider type code, and dates of service with ICD-9/10-CM diagnosis, ICD-9-CM/ICD-10-Procedure Coding System (PCS) procedure codes, and CPT-4 codes. Data were linked based upon Medicaid identifier or, if Medicaid identifier was not available, a ‘fuzzy match’ process with name, date of birth, zip code and phone number was used. Once linked, OSEDA stripped all HIPAA identifiers from the data, retaining only the previously generated random number ID, and transferred this linked file to investigators at Washington University. There were 6402 women in this linked dataset (91% of whom were successfully linked via Medicaid identifier). Address was separately transferred to investigators in conjunction with the randomly generated ID for geographic analyses. No clinical information attached to HIPAA identifiers was transferred between entities, minimizing the risk to confidentiality. Figure 2 illustrates the flow of data from different sources.

Medicaid claims were utilized to most accurately define the delivery date as the provider or facility date associated with a CPT-4 or ICD-9/10-CM or PCS code for delivery. In order to identify this date, a hierarchical algorithm was implemented.: 1) facility and provider claims with CPT-4 or ICD 9/10 code for delivery during an inpatient admission; 2) CPT-4 or ICD9/10 code for delivery and an additional code for delivery-related diagnosis or procedure; 3) CPT-4 or ICD 9/10 code for delivery and a provider claim for anesthesia/epidural; 4) CPT-4 or ICD9/10 code for delivery and a provider claim for postpartum care following birth; or 5) 3 or more delivery-specific codes (including pathologic examination of the placenta and revenue code for labor room (720–724, 729)). The majority of delivery dates (86%) were identified using the first 2 steps in the algorithm. Of the 6402 women in the linked dataset, the full algorithm identified at least one delivery date for 5040 women. For delivery identification codes, (see Additional file 1: Table S1 and Additional file 2: Table S2). This procedure mirrored a previously validated algorithm for identifying deliveries using administrative data [15].

The conception date was defined as 280 days (40 weeks) prior to the delivery date, based upon the standard gestational period. The time frame between the conception date and delivery date was used to identify gestational diabetes and pregnancy comorbidities and exclude pre-existing diabetes. The time before the conception date was used to identify pre-existing diabetes for exclusion as well. The strictest definition was applied so that a woman was only included in the final population as having probable gestational diabetes if she had an ICD-9/10-CM diagnosis code for gestational diabetes during the period from conception to delivery or a documented laboratory diagnosis by Carpenter and Coustan criteria on a 3 h oral glucose tolerance test during pregnancy (*n* = 1078). These are standard criteria for diagnosis of gestational diabetes if 2 or more of the following conditions are true: fasting glucose > 5.27 mmol/L, 1 h glucose > 9.99 mmol/L, 2 h glucose > 8.60 mmol/L, 3 h glucose > 7.77 mmol/L. Figure 1 further delineates inclusion and exclusion criteria. The first birth per woman coded for gestational diabetes with no evidence of pre-existing diabetes served as the index delivery. As the primary outcome was receipt of recommended screening by 12 weeks postpartum, individuals with deliveries after October 8, 2015 were excluded as they would not have had the full opportunity to achieve the primary outcome.

For the final population, demographic data including age at delivery, race, ethnicity, and preferred language were available from EHR records. Height and weight to calculate BMI prior to the index delivery were missing on the majority of the population and are not reported. Home address from EHR records was geocoded using ESRI ArcGIS (Redlands, CA). This information was then associated with a census tract and linked to census tract level variables. These variables included modified retail food environment index (mRFEI), Rural Urban Continuum Codes (RUCC), and access to public transportation. The mRFEI is a measure developed by the Centers for Disease Control and Prevention (CDC) in 2011 and calculated as follows: 100* (healthy retailers/(unhealthy retailers + healthy retailers)) [16]. Higher scores on this scale represent better access to healthy food. This index classified healthy retailers as supermarkets and large grocery stores, fruit and vegetable markets, and warehouse clubs while unhealthy retailers included small grocery stores, convenience stores, and fast food restaurants. The RUCC from 2013 are defined by census tract and are publicly available [17]. Classification ranges from 1 to 9, defining census tracts according to metropolitan, non-metropolitan urban, and rural areas by population. Individuals were considered to live near public transportation if they lived in a city with bus and/or train routes, as determined by their zip code of residence. The driving distance in kilometers from a woman’s home to nearest health center was also calculated.

Pregnancy comorbidities and delivery complications were defined exclusively using Medicaid claims data as this information was incomplete in EHR records. Specific administrative codes were used to identify each comorbidity or complication (see Additional file 1: Table S1). A pregnancy-specific comorbidity index was calculated using a previously validated algorithm [18]. Healthcare utilization metrics including number of prenatal visits to a healthcare facility, prenatal visits to a certified diabetes educator (CDE), and number of postpartum visits to a healthcare facility were calculated utilizing EHR encounters and CPT-4 codes from the Medicaid claims data. Completion of screening tests for diabetes in the postpartum period was recorded by type of test and time frame. These tests were identified utilizing lab names and results from the EHR or CPT-4 codes from Medicaid claims data. Screening rates were defined as a percent of the final population (*n* = 1078) for FPG, oGTT, HbA1C, and any glucose test within 12 weeks of delivery and from 12 weeks to 1 year postpartum. Screening rates for recommended tests (FPG or oGTT within 12 weeks postpartum and FPG, oGTT, HbA1C after 12 weeks postpartum) were also reported. As some women received multiple screening tests, the unique number and percent of women screened within the first postpartum year and at any point after delivery were also described. Among those who had more than 1 year of postpartum data (*n* = 672), the number and percent of women receiving a first screening test more than 1 year after delivery were calculated. All data management procedures and analyses were conducted using SAS Enterprise Guide v. 7.1 (Cary, NC).

## Results

The median age at delivery for women in the final gestational diabetes population (*n* = 1078) was 28 (IQR 24–33) years, and 19.6% of the population was advanced maternal age (> 35 at delivery). Self-reported racial or ethnic minorities comprised over half of the population with 40.6% of women identifying as black non-Hispanic. Preferred language other than English was reported in 17.9% of the population (with an additional 12.3% not reporting a preferred language), reflecting the large immigrant and refugee populations served by some of the health centers. The majority of the population (69.2%) lived in a metropolitan area with a population > one million, with only 13% living in areas with a population under 20,000 people. The median distance from home to the closest health center was 4.7 km (IQR 2.2–10.4), and 69.5% percent of the population lived near public transportation. There were no healthy food retailers according to the mRFEI in the census tract of residence for 18.9% of the population.

In our final population, 72.1% of the women had a comorbidity index of 1 or higher and 15.6% had a comorbidity index of 4 or higher. This pregnancy specific comorbidity index was initially developed in an external cohort of women on Medicaid in pregnancy. In that cohort, less than 50% of the population had a score of 1 or higher and each additional point increased the risk of maternal end organ injury or death within 30 days of delivery by 37% (95% CI 35–39%) [18]. Hence, our population was relatively complicated for a pregnant population. Pre-existing or transient gestational hypertension were among the most common comorbidities, affecting 21% of the population together. Additionally, 10.5% of women had mild or severe pre-eclampsia. One or more codes for depression were present in 21.4% of the population before or after delivery. In regard to delivery complications, 23.9% of women had a Cesarean section in a prior pregnancy and 34.2% of women had a Cesarean section to deliver the index pregnancy. Table 1 summarizes demographics, comorbidities, and pregnancy and delivery complications.

**Table 1:** Demographics, geographic data, and clinical characteristics of low income women with gestational diabetes in Missouri (*n* = 1078)

Demographics	Median (IQR) or n(%)
Age at delivery	28 (24–33)
Advanced maternal age (35 + at delivery)	211 (19.6)
Race/ethnicity	
White non-Hispanic (or ethnicity unreported)	338 (31.4)
Black non-Hispanic (or ethnicity unreported)	438 (40.6)
Hispanic	168 (15.6)
Asian	45 (4.2)
Other (Native American, Pacific Islander, More than one Race)	37 (3.4)
Missing/Unreported	52 (4.8)
Preferred Language other than English	193 (17.9)
Preferred Language not available	133 (12.3)
Geographic data^a^	
Residence in county with metropolitan area > one million (*n* = 1044)	722 (69.2)
Lived near public transportation (*n* = 1063)	739 (69.5)
Distance from home to nearest health center (km) (*n* = 1041)	4.7 (2.2–10.4)
Modified retail food environment index (mRFEI) by census tract^b^ (*n* = 1053)	9.1 (4.8–14.3)
Census tract of residence had no healthy food retailers by mRFEI (n = 1053)	199 (18.9)
Selected comorbidities and pregnancy complications^c^	
Pregnancy specific comorbidity index ≥ 1^d^	777 (72.1)
Drug abuse	55 (5.1)
Pre-existing depression	186 (17.3)
Postpartum depression without pre-existing depression	45 (4.2)
Asthma	95 (8.8)
Pre-existing hypertension	153 (14.2)
Transient gestational hypertension w/o pre-existing hypertension	73 (6.8)
Mild pre-eclampsia	63 (5.8)
Severe pre-eclampsia/Eclampsia	50 (4.6)
Selected delivery complications^e^	
C-section (prior pregnancy)	258 (23.9)
C-section (this pregnancy)	369 (34.2)
Induction	340 (31.5)
Cord complication	140 (13.0)
Chorioamnionitis	112 (10.4)
Preterm labor	80 (7.4)
Abnormal forces of labor	66 (6.1)
Malpresentation	67 (6.2)
Obstruction of labor without dystocia	48 (4.5)
Shoulder dystocia	40 (3.7)

^a^Missing geographic data resulted from inability to match to home address to geocode, out of state address listed, or missing clinic information

^b^ Per the CDC, mean mRFEI in Missouri and nationally is 10

^c^ Other comorbidities present in less than 5% of the population: congenital heart disease, congestive heart failure, ischemic heart disease, human immunodeficiency virus (HIV), alcohol abuse, pulmonary hypertension, chronic renal disease, sickle cell disease, systemic lupus erythematosus, cardiac valve disease, multiple gestation

^d^ In the validation cohort for the comorbidity index, developed in a Medicaid population, less than half the population had a score of 1 or higher. Each additional point increase in comorbidity index was associated with a 37% increase (95% CI 1.35–1.39) in maternal end organ injury or death from delivery to 30 days postpartum

^e^ Other delivery complications present in less than 5% of the population: fetopelvic disproportion, postpartum hemorrhage, placenta previa, placental abruption, other delivery infection, hypotension, severe laceration

We next address access to care and adherence to screening guidelines. Seventy three percent of women had a visit to a healthcare facility (documented in either the EHR or Medicaid claims files) in the first 12 weeks postpartum, and 83.9% had a visit within the first postpartum year. The median number of prenatal visits documented in either the EHR or Medicaid claims files was 11.5 (IQR 7–16), and the median number of postpartum visits was 3 (IQR 1–5). Visits in both the prenatal and postpartum time frames could have included visits with a nurse, nurse practitioner, physician’s assistant, or physician, as documentation of billing provider in both Medicaid and EHR data was incomplete. Ten percent of the population was on an oral hypoglycemic medication during pregnancy and 2.5% of women were on insulin. For the protocol used to identify medication type in EHR data, (see Additional file 3: Table S3). Twelve percent of women received at least one prenatal visit with a certified diabetes educator. Twenty one percent of the population had a subsequent pregnancy during the study period. Median follow-up for the final population until subsequent pregnancy conception date or last visit date in either EHR or Medicaid claim files was 1.2 years (IQR 0.3–2.5). For those with more than 1 year of data (*n* = 672), median follow-up time until next pregnancy conception date or last visit date was 2.1 years (IQR 1.3–3.3). Table 2 details healthcare utilization in the final gestational diabetes population.

**Table 2:** Healthcare utilization among low income women with gestational diabetes in Missouri (*n* = 1078)

Prenatal healthcare utilization	Median (IQR) or n (%)
Oral hypoglycemic medicine prescribed during pregnancy	108 (10.0)
Insulin prescribed during pregnancy	27 (2.5)
Total visits during prenatal period ^a^	11.5 (7–16)
Time from first prenatal visit to delivery (weeks) (*n* = 1065)	29.7 (23.0–33.1)
Women with ≥ one prenatal CDE visits	130 (12.1)
Women with no medical records available prior to prenatal record	395 (36.6)
Postpartum maternal healthcare utilization^b^	Median (IQR) or n (%)
Total visits within one year postpartum ^a^	3 (1–5)
Women with at least one visit in first 12 weeks postpartum ^a^	787 (73.0)
Women with at least one visit in the first postpartum year ^a^	904 (83.9)
Postpartum data available for ≥ one year	672 (62.3)
Length of follow-up for whole population (years) ^c^	1.2 (0.3–2.5)
Length of follow-up for individuals with > one year of data (years) ^c^	2.1 (1.3–3.3)

^a^ These include any visit to a healthcare facility present in either the Medicaid file, EHR file, or both. These can include nurse visits, but do not include CDE visits or visits with a registered dietician (RD)

^b^ These are maternal care visits only (newborn care was not linked). If another pregnancy occurred within the first postpartum year, this represents postpartum visits prior to conception date for next pregnancy

^c^ Time in years between delivery of index pregnancy and estimated conception date for next pregnancy or last visit date

Table 3 depicts screening rates by type of test and time frame. For the primary outcome, 105 women (9.7% of the total population) received a recommended test (FPG or oGTT) to screen for type 2 diabetes by 12 weeks postpartum. By 1 year postpartum, 204 women (18.9% of the total population) had received a recommended test (HbA1C (if done after 12 weeks), FPG, or oGTT). Of the whole population, 329 women (30.5%) received at least one recommended screening test during the study period and before a subsequent pregnancy. After 12 weeks postpartum, the most common recommended screening test was an HbA1C. At all times points, it was more common to have any glucose test completed than it was to have a recommended test. Any glucose test could include a random blood or urine glucose or a glucose on a complete or basic metabolic panel or renal function panel. More than 1 year of follow-up data were available on a subset of women (*n* = 672). In this group, 125 women (18.6% of the subset) completed a first recommended screening test more than 1 year after the index delivery.

**Table 3:** Postpartum diabetes screening rates among low income women with gestational diabetes in Missouri

	FPG ^a^ n (%)	2hoGTT ^a^ n (%)	HbA1C ^a^ n (%)	Recommended test^b^ n (%)	Any glucose test^c^ n (%)
Whole Population (n = 1078)					
Screened 0–12 weeks postpartum^d^	39 (3.6)	78 (7.2)	49 (4.6)	105 (9.7)	293 (27.2)
Screened 12 weeks- one year postpartum ^d^	16 (1.5)	15 (1.4)	107 (9.9)	121 (11.2)	246 (22.8)
Screened by one year postpartum (unique)	55 (5.1)	92 (8.5)	141 (13.1)	204 (18.9)	438 (40.6)
Screened at any time from delivery to next pregnancy/last follow-up (unique)	75 (6.9)	107 (9.9)	272 (25.2)	329 (30.5)	575 (53.3)
Women with > one year follow-up data (n = 672)					
1st screening test done >one year postpartum	20 (3.0)	15 (2.2)	131 (19.5)	125 (18.6)	137 (20.4)

^a^FPG = fasting plasma glucose; 2hoGTT = 2 h oral glucose tolerance test; HbA1C = hemoglobin A1C

^b^ Recommended = FPG or 2hoGTT in first 12 weeks; FPG, oGTT, or HbA1C after 12 weeks

^c^ Includes random blood or urine glucose, complete metabolic panel, basic metabolic panel, renal function panel, and all recommended tests

^d^ There are some women who were screened both within 12 weeks and between 12 weeks and 1 year postpartum

Given the possibility of loss of Medicaid coverage after 60 days postpartum, we examined how screening tests were identified in different time frames (CPT codes came from Medicaid claims and laboratory data came from EHR files). In the first 12 weeks postpartum, it was more common for labs to be identified by CPT code than EHR laboratory data. After 12 weeks postpartum, it was more common for tests to be identified with EHR laboratory data than CPT code data. For the CPT codes used to identify visits and screening tests, (see Additional file 2: Table S2).

## Discussion

To our knowledge, this is the first time that statewide EHR data from FQHCs has been linked to Medicaid administrative claims data to provide population estimates of follow-up care among women with gestational diabetes covered by Medicaid during pregnancy. This linked dataset allows us to more comprehensively study a population that is understudied because of fragmentation of care and loss of health insurance after pregnancy. During the study period, Medicaid income eligibility in Missouri was 185–200% of the federal poverty level (FPL) during pregnancy and 22–37% of the FPL for parents after pregnancy [19]. Utilizing the FQHC EHR data allowed us to continue to follow these women after they were no longer covered by Medicaid. Likewise, the Medicaid claims data provided comprehensive delivery information that was unavailable in the clinic record. Through leveraging the strengths of each data source, we present a more complete picture of healthcare for these women. Given that all FQHCs have to report some data to the federal government for the Uniform Data Set, it is possible that this analysis could be replicated in other states through the data vendors for their primary care associations.

Our population is representative of women in Missouri receiving care in FQHCs on Medicaid during pregnancy and as free from bias as possible given the study design and data sources. When compared with publicly available information from the Uniform Data Set, for the centers that consented to use of their data, the number of women included in our population from each health center was generally proportional to the number of prenatal patients delivered at each center during the study period. The racial and ethnic composition of our population was comparable to that expected from publicly reported data on the patient populations served at the included health centers.

We demonstrated higher screening rates for women receiving Medicaid during pregnancy than exhibited in analyses that used administrative claims data alone [7, 8]. We found that 9.7% of our population received a recommended screening test by 12 weeks postpartum (8.4% if restricted to 4–12 weeks postpartum), as compared with 3.2% in South Carolina Medicaid claims. Twenty seven percent of our population had any glucose test at 12 weeks and 40.6% had any glucose test by 1 year postpartum, as compared with 5.7 and 15.2% in a Maryland Medicaid claims population. Moreover, 18.9% of our population had received a recommended test by 1 year postpartum, and 30.5% received a recommended screening test at any point after delivery and before a subsequent pregnancy. While this is lower than screening rates reported in privately insured populations and integrated health systems, [10, 20, 21] our population’s ability to receive optimal follow-up care is complicated by challenges with healthcare access, fragmented care delivery, and other social determinants of health.

Our results show that there are clear opportunities for increasing glucose screening in this population. A majority of women had a visit to a healthcare facility in the postpartum period (73% by 12 weeks and 83.9% by 1 year). Even if we assume that many of the women who had a visit received an order for screening that was not completed, the gap between the number of women with a recommended test and the number of women with any glucose test represents a window of opportunity for increasing screening. Twice as many women received any glucose test (22.8%) as received a recommended test (11.2%) between 12 weeks and 1 year postpartum. As women are getting labs completed for other purposes, and the HbA1C is a non-fasting test, it is possible to increase screening through this modality in the time frame after 12 weeks postpartum.

Another strength of our population is the racial and ethnic diversity, with 40.6% black non-Hispanic women. In many other large studies of postpartum screening, black women have traditionally been under-represented as compared to their proportion of the US population and other racial and ethnic groups [10, 12, 21–25]. Our study adds additional screening data to the literature in this group at high risk for progression to type 2 diabetes [4]. Further, our study reports on screening that occurs more than 1 year after delivery, which is unique to the literature. This is important because it appears that women continue to receive screening after 1 year postpartum, even if they have not been previously screened. Women may be more able to prioritize their health after adapting to the demands of childcare in the first year. Some of these later screening tests may occur incidentally when women present for other healthcare needs. Finally, we were able to incorporate geographic data on our population, including distance from home to nearest clinic, neighborhood food environment, and proximity of public transportation. These factors may affect follow-up care, diabetes screening and diabetes prevention but have not been examined in prior literature.

Our study has a number of limitations. First, we were only able to assess completed or billed laboratory tests and did not have data on all ordered tests. If a laboratory test was not mapped in the central data warehouse or not billed to Medicaid, we would not have captured it. However, we were able to capture at least some point of care glucose and HbA1C measures, in addition to traditional labs, in the central data warehouse. Further, while we could capture screening billed to Medicaid from all sites (FQHC and other) while a woman was still receiving Medicaid, screening after Medicaid lapsed was only available through our FQHC sites. However, as our FQHCs provide essential care for uninsured women and our population was initially identified as receiving care in FQHCs, it is a reasonable assumption that the majority of care for our population would continue to occur in this setting.

We also had no data prior to the prenatal record for 36.6% of women. This is not surprising given that preventive care is often foregone among patients with few resources. Additionally, for 119 women who delivered before October 10, 2010, we did not have the opportunity for a full 280 day prenatal care record. Hence, we may have missed comorbidities or prenatal visits in these women. For the 164 women that delivered in 2015, we did not have the opportunity to capture a full postpartum year and may have missed postpartum screening between 12 weeks and 1 year in this subset; however, excluding these women did not affect screening estimates substantially. For prenatal CDE visits, we were only able to document billed visits. If these visits were not billed by the health center or if the health center provided diabetes education with a non-CDE provider, this would not be captured. This is important to note as many health centers are not able to maintain a CDE on staff. While we have identified visits to a healthcare facility in both prenatal and postpartum time frames, it was difficult to differentiate between nurse visits and visits with a doctor, nurse practitioner, or physician’s assistant. We did assess the Medicaid claims files for visits that had CPT codes that were level 3 or higher (99203-99205 or 99213-99215) as these are less likely to be nurse-only visits. In the prenatal time frame, the median number of level 3 or higher visits was 5 (IQR 2–9). Level 3 or higher visits were very infrequent in the postpartum time period. Given bundled billing for prenatal and postpartum care, this method likely under-counts visits with a provider.

There were three FQHC systems serving predominantly rural populations that did not approve release of their data, leading to over-representation of urban areas in our study population. Additionally, if some centers were better at coding gestational diabetes diagnoses and having laboratory data sent to the centralized data warehouse, we were not able to eliminate that potential bias. While we sought to incorporate clinical data where possible, height, weight, and BMI were available on less than half of the women and are not reported. We utilized administrative codes to identify comorbidities and complications as well as some of the laboratory tests completed for screening. This limitation is mitigated by the fact that many of these codes have been validated in prior studies [15, 18, 26–30]. Medication and supply data were available from the EHR, but not in our administrative claims data. We may have missed oral hypoglycemic therapy or insulin that was added to a patient’s regimen if care was transferred from an FQHC to a specialty center during pregnancy. However, published data suggest that 70–85% of women with gestational diabetes can be managed without medication, so our finding of 12.5% of our population on medication during pregnancy may not underestimate medication use substantially [5]. Finally, while our data is only a subset of the Missouri Medicaid population with gestational diabetes during this time frame, there is a depth of information available in this population that is absent from previously published work.

## Conclusions

By utilizing EHR data linked to Medicaid claims data, we have documented that screening for type 2 diabetes after a pregnancy with gestational diabetes occurred in our predominately urban population at rates higher than previously reported in Medicaid claims data alone. However, there are opportunities for increasing screening at all time points. Additionally, documentation of screening after the first postpartum year is important as the risk for developing type 2 diabetes is lifelong. Further analysis of this data will examine individual, health system, and societal factors associated with lack of screening to inform interventions that close the gap between current practice and guideline recommended screening.

## Additional files

Additional file 1: **Table S1.** Conditions and associated ICD9/10-CM Diagnosis or procedure codes. (PDF 32 kb)

Additional file 2: **Table S2.** Procedures, Visits, and Laboratory tests and associated CPT codes. (PDF 14 kb)

Additional file 3: **Table S3.** Medication names used in identifying types of diabetes prescriptions. (PDF 8 kb)

## Abbreviations

ACOG: American Congress of Obstetricians and Gynecologists
ADA: American Diabetes Association
CDC: Centers for Disease Control and Prevention
CDE: Certified diabetes educator
CI: Confidence interval
CPT-4: Current Procedural Terminology, 4th edition
EHR: Electronic health record
FPG: Fasting plasma glucose
FPL: Federal poverty level
FQHC: Federally Qualified Health Center
HbA1C: Hemoglobin A1C
HIV: Human immunodeficiency virus
ICD/10-CM: International Classification of Diseases, Clinical Modifications versions 9 or 10 diagnosis code
ICD-9/10-PCS: International Classification of Diseases, Clinical Modifications versions 9 or 10 Procedure Coding System
IQR: Interquartile range
MPCA: Missouri Primary Care Association
mRFEI: Modified retail food environment index
oGTT: Oral glucose tolerance test
OSEDA: Office of Social and Economic Data Analysis
RD: Registered dietician
RUCC: Rural Urban Continuum Codes
