# Supplementary material for: Postpartum diabetes screening among low income women with gestational diabetes in Missouri 2010–2015
Source: BMC Public Health. 2019 Feb 4;19:148. doi: 10.1186/s12889-019-6475-0 (PMC6360751; doi:10.1186/s12889-019-6475-0)
Supplement: Supplementary file 1 — Table S1. Conditions and associated ICD9/10-CM Diagnosis or procedure codes. (PDF 32 kb) [file 12889_2019_6475_MOESM1_ESM.pdf]

Supplementary Table S1- Conditions and associated ICD9/10-CM Diagnosis or Procedure Codes

Condition	ICD-9-CM Diagnosis or Procedure Code	ICD-10-CM-Diagnosis or Procedure Code
Delivery identification		
Any delivery	V27, 650<br>72xx 73xx 740-742 744 7499	Z37 O80 O82<br>0Q820ZZ 0Q823ZZ 0Q824ZZ 0Q830ZZ 0Q833ZZ 0Q834ZZ 0U7C7ZZ 0W8NXZZ 10900ZC 10903ZC 10904ZC 10907ZA 10907ZC 10908ZA 10908ZC 10A07ZZ 10A08ZZ 10D00Z0 10D00Z1 10D00Z2 10D07Z3 10D07Z4 10D07Z5 10D07Z6 10D07Z7 10D07Z8 10E0XZZ 10J07ZZ 10S07ZZ 10S0XZZ 3E030VJ 3E033VJ 3E040VJ 3E043VJ 3E053VJ 3E060VJ 3E063VJ 3E0P7GC
Diabetes Identification		
Gestational diabetes	6488 6480 (only if noted after 6488)	O244
Pre-existing diabetes mellitus	249 250 6480 (if not after 6488)	O240 O241 O243 O248 O249 E08 E09 E10 E11 E13
Type 2 diabetes	250x0 250x2	E11
Comorbidities		
Mild pre-eclampsia	6424	O11 O140
Severe pre-eclampsia	64251 64252 64253 64254 64261 64262 64263 64264	O141 O142 O150 O151 O152 O159
Pre-existing hypertension	64200 64201 64202 64203 64204 64211 64212 64213 64214 64221 64222 64223 64224 64271 64272 64273 64274 4010 4011 4012 4013 4014 4015 4016 4017 4018 4019 4020 4021 4022 4023 4024 4025 4026 4027 4028 4029 4030 4031 4032 4033 4034 4035 4036 4037 4038 4039 4040 4041 4042 4043 4044 4045 4046 4047 4048 4049 4050 4051 4052 4053 4054 4055 4056 4057 4058 4059	I10 I11 I12 I13 I15 O10 O11
Transient Gestational hypertension	64230 64231 64232 64233 64234	O161 O162 O163 O164 O165 O169 O131 O132 O133 O134 O135 O139
Depression	29620 29621 29622 29623 29624 29625 29630 29631 29632 29633 29634 29635 2965 2966 29682 29690 3004 3090 3091 311<br>30928	Z373 Z3760 Z3761 Z3762 Z3763 Z3764 Z3769 Z377 F320 F321 F322 F323 F324 F325 F328 F329 F330 F331 F332 F333 F338 F339 F341 F348 F349 F412 F3131 F3132 F314 F315 F316 F348 F349 F380 F381 F388 F39 F99
Alcohol abuse	291 303 3050	F10
Drug abuse	304 3052 3053 3054 3055 3056 3057 3058 3059 6483	F11 F12 F13 F14 F15 F16 F18 F19
Asthma	493	J44 J45
Chronic ischemic heart disease	412 413 414	I20 I25
Congenital heart disease	7450 7474 6485	Q20 Q21 Q22 Q23 Q24 Q25 Q26 O994
Congestive heart failure	42822 42823 42832 42833 42842 42843	I5022 I5023 I5032 I5033 I5042 I5043
HIV	042 V08	B20 B24 O987 Z21
Pulmonary hypertension	4160 4168 4169	I270 I272 I278 I279
Renal disease	581 582 583 585 587 588 6462	N022 N03 N04 N05 N08 N171 N172 N18 N25 O2683

Sickle-cell disease	2824 2826	D56 D57
Systemic lupus erythematosus	7100	M32
Valve disease	394 395 396 397 424	I05 I06 I07 I08 I09 I34 I35 I36 I37 I38 I39
Infant characteristics		
Multiple gestation	V272 V273 V274 V275 V276 V277 651	Z372 Z373 Z374 Z377 Z3750 Z3751 Z3752 Z3753 Z3754 Z3759 Z3760 Z3761 Z3762 Z3763 Z3764 Z3769 O30 O31
Large for gestational age	65660 65661 65663	O3661X0-O3661X9<br>O3662X0-O3662X9<br>O3663X0-O3663X9<br>O3660X0-O3660X9
Small for gestational age	65650 65651 65653	O365110-O365119<br>O365120-O365129<br>O365130-O365139<br>O365910-O365919<br>O365920-O365929<br>O365930-O365939<br>O365190-O365199<br>O365990-O365999
Delivery complications		
Abnormal forces of labor	66101 66111 66121 66141 66191 66201 66211 66221 66231	O620 O621 O622 O624 O628 O629 O631 O639 O630 O632
Caesarean section	6542 (previous)<br>740 741 742 744 7499	O3421 (previous)<br>10D00Z0 10D00Z1 10D00Z2
Chorioamnionitis	65841	O411010- O411019<br>O411020-O411029<br>O411030-O411039<br>O411210 -O411219<br>O411220 -O411229<br>O411230- O411239<br>O411410 -O411419<br>O411420 -O411429<br>O411430 -O411439<br>O411090- O411099<br>O411290- O411299<br>O411490- O411499
Cord complication	66301 66311 66321 66331 66341 66351 66361 66381 66391	O43121 O43122 O43123 O43129<br>O690XX0 -O690XX9<br>O691XX0 -O691XX9<br>O692XX0- O692XX9<br>O693XX0- O693XX9<br>O695XX0 -O695XX9<br>O6981X0 -O6981X9<br>O6982X0- O6982X9<br>O6989X0-O6989X9<br>O694XX0 -O694XX9<br>O699XX0- O699XX9
Fetopelvic disproportion	65301 65311 65321 65331 65341 65351 65361 65371 65381 65391 66011 66021	O331 O334XX0-O334XX9<br>O335XX0-O335XX9<br>O337 O338 O339 O651 O654 O662 O330 O332 O333XX0-O333XX9 O336XX0-O336XX9 O338 O339 O650 O651 O652 O653 O654 O655 O658 O659
Hypotension	66911 66912 66913 66914 66921 66922 66923 66924	O2650 O2651 O2652 O2653 O751
Induction of labor	65901 65911<br>7301 7309 731 734	O610 O611 O618 O619<br>0U7C7ZZ 10900ZC 10903ZC 10904ZC 10907ZC 10908ZC 10907ZC 10908ZC 3E030VJ 3E033VJ 3E040VJ 3E043VJ

		3E050VJ 3E053VJ 3E060VJ 3E063VJ 3E0DXGC 3E0P7GC
Infection during delivery	6700 6701 6702 6703 6704 6705 6706 6707 6708 6720 65931	O753 O8612 O864 O85
Malpresentation	65201 65211 65221 65231 65241 65251 65261 65271 65281 65291	O320XX0-O320XX9<br>O321XX0-O321XX9<br>O322XX0-O322XX9<br>O323XX0-O323XX9<br>O324XX0-O324XX9<br>O326XX0-O326XX9<br>O328XX0-O328XX9<br>O329XX0-O329XX9<br>O641XX0-O641XX9<br>O642XX0-O642XX9<br>O643XX0-O643XX9<br>O644XX0-O644XX9<br>O645XX0-O645XX9<br>O648XX0-O648XX9<br>O649XX1-O649XX9<br>O661 O640XX0-O640XX9 O660
Obstruction of labor	66001 66011 66021 66031 66041 66051 66061 66071 66081 66091	O640XX0-O640XX9<br>O641XX0-O641XX9<br>O642XX0-O642XX9<br>O643XX0-O643XX9<br>O644XX0-O644XX9<br>O645XX0-O645XX9<br>O648XX0-O648XX9<br>O649XX0-O649XX9 O650 O651 O652 O653 O654 O655 O658 O659 O660 O662 O663 O6640 O6641 O666 O668 O669 O661
Placental abruption	64120 64121 64123	O458X1 O458X2 O458X3 O4591 O4592 O4593 O45001 O45002 O45003 O45009 O45011 O45012 O45013 O45019 O45021 O45022 O45023 O45029 O45091 O45092 O45093 O45099 O458X9 O4590
Placenta previa	6410 6411	O44
Post-partum hemorrhage	6660 6661 6662 6663 9904 9905	O720 O721 O722 O723
Preterm labor	64421	O6012X0-O6012X9<br>O6013X0-O6013X9<br>O6014X0-O6014X9<br>O6022X0-O6022X9<br>O6023X0-O6023X9<br>O6010X0-O6010X9<br>O6020X0-O6020X9
Severe laceration	66421 66431<br>7562	O702 O703<br>0DQP0ZZ 0DQP3ZZ 0DQP4ZZ 0DQP7ZZ 0DQP8ZZ 0DQQ0ZZ 0DQQ3ZZ 0DQQ4ZZ 0DQQ7ZZ 0DQQ8ZZ 0DQR0ZZ 0DQR3ZZ 0DQR4ZZ
Shoulder dystocia	66041	O660
Healthcare utilization		
Post-partum care	V24	
Prenatal care	V22 V23 V28	
