# Supplementary material for: Postpartum diabetes screening among low income women with gestational diabetes in Missouri 2010–2015
Source: BMC Public Health. 2019 Feb 4;19:148. doi: 10.1186/s12889-019-6475-0 (PMC6360751; doi:10.1186/s12889-019-6475-0)
Supplement: Supplementary file 2 — Table S2. Procedures, Visits, and Laboratory tests and associated CPT codes. (PDF 14 kb) [file 12889_2019_6475_MOESM2_ESM.pdf]

Supplementary Table S2- Procedures, Visits, and Laboratory tests and associated CPT codes

Condition	CPT Code	Notes
Laboratory tests		
Hemoglobin A1C	83036 83037	
50G Glucose Challenge test	82950	
Fasting Glucose	82947	
Fasting, 1HR, 2HR Glucose	82951	2h oGTT could be billed 82951 alone or 82947+82950
3HR Glucose	82952	3h oGTT is billed as 82951+82952
Any Glucose Test	80047 80048 80050 80053 80069 82947 82950 82951 82952 82962 83036 83037	
Healthcare utilization		
Certified Diabetes Educator Visit	G0108 G0109	
Registered Dietician Visit	97802-97804 G0270 G0271	
Office visit	99201-99205 99211-99215	
Delivery identification		
Delivery	59400 59409 59410 59412 59414 59510 59514 59515 59525 59610 59612 59614 59618 59620 59622	incorporates bundled prenatal and postpartum care
Surgical pathology examination	88300 88307	of placenta
Anesthesia	01960 01961 01968	
Epidural	01967	
