# Supplementary material for: Postpartum diabetes screening among low income women with gestational diabetes in Missouri 2010–2015
Source: BMC Public Health. 2019 Feb 4;19:148. doi: 10.1186/s12889-019-6475-0 (PMC6360751; doi:10.1186/s12889-019-6475-0)
Supplement: Supplementary file 3 — Table S3. Medication names used in identifying types of diabetes prescriptions. (PDF 8 kb) [file 12889_2019_6475_MOESM3_ESM.pdf]

Supplementary Table S3- Medication names used in identifying types of diabetes prescriptions

Type of Medication	Medications*
Insulin	Humulin, Novolin, Relion, Humalog, Novolog, Apidra, Levemir, Lantus, Toujeo, solostar, insulin [aspart, detemir, glargine, regular, pump]
Oral	amaryl diabeta glimepiride glipizide glucophage glucotrol glucovance glumetza glyburide metformin

*Fuzzy match algorithm matching parts of medication name used to identify the medications. Each insulin name encompasses all variations of that brand. Each oral medication encompasses different formulations including extended release and combination therapy and includes only classes typically prescribed in pregnancy.
